# Open Source Seed, a Revolution in Breeding or Yet Another Attack on the Breeder’s Exemption?

**DOI:** 10.3389/fpls.2019.01127

**Published:** 2019-09-18

**Authors:** Niels Louwaars

**Affiliations:** ^1^Director, Plantum, Gouda, Netherlands; ^2^Department of Law and Governance, Wageningen University, Wageningen, Netherlands

**Keywords:** open source, Intellectual Property, Nagoya Protocol, seed system interventions, breeders rights

## Abstract

The Open Source Seed Initiative was initiated in 2012. Following concerns about the concentration in the seed sector and the rise of patenting, the initiative is “dedicated to maintaining fair and open access to plant genetic resources worldwide in order to ensure the availability of germplasm to farmers, gardeners, breeders, and communities of this and future generations.” Inspired by the debate on the anti-commons and the open source software movement, the initiative wants to create a viral system to “free” genetic resources: the use of “freed” genetic resources is made conditional to any materials derived from them being made available under the same “open source” conditions. This would be achieved under a “pledge” (in the USA) or a license contract (in Germany). The objective of this paper is to analyze whether these open source seed initiatives may deliver their goals. We compare the concept with the open innovation character of the plant breeder’s rights system, exemplified by the breeder’s exemption, and the major other open source initiative in the sector, BiOS. We also present other ways to limit negative impact of the patent system on plant breeding. We conclude that national sovereign rights on genetic resources may challenge the open source goals and that the German initiative may contribute to legal complexities in the seed sector. The open source movement may even contribute to the trend that openness (through the breeder’s exemption) is challenged despite the intentions to the contrary. In fact, the initiatives not only free the genetic resource but also treat seeds as a common good. We question the sustainability of the business models for that approach and thus the societal benefits that can be expected from plant breeding, which may illustrate the tragedy of the commons.

## Introduction

Intellectual property rights (IPR) systems have been developed in order to stimulate innovation that will serve society. Providing exclusive rights to inventors and authors provides both recognition and a basis for right holders to commercialize their intellectual assets, i.e., that users share benefits with them. The basic argument for society to grant such rather monopolistic rights is that society gets something in return. Patent applicants have to describe their invention in such a way that someone “skilled in the art” can rework it; the rights are time-bound, which means that the invention or works of art will be in the public domain at some stage. Similar to property rights at large, an important argument for such rights is the “tragedy of the commons” (Hardin, 1968), meaning that resources are underutilized or insufficiently cared for when all have access to them and nobody takes responsibility.

Intellectual property rights were introduced in plant breeding quite recently. Apart from the Plant Patent in the USA, which is available to new varieties of many (not all) vegetatively propagated crops, such rights emerged in Europe only in the 1960s when the concept of Plant breeder’s rights was introduced in a number of countries. This which spread around the world was supported by the TRIPS agreement of the World Trade Organization (WTO). Such rights relate to plant varieties only, the totality of characteristics of a certain well described group within a species. Patents on individual traits and biotechnologies entered the breeding sector only during the past three decades.

Since IPR systems should create a “quid pro quo”—a balance between the rights and the benefits for society, discussions are multiple about whether the systems (still) do that, especially when new technologies emerge such as information—and biotechnologies. The operation of IPR protection systems is under public scrutiny in various industries. Strong IPRs are claimed to cause high medicine prices, concentration of power in the Information Technology, and publishing sectors. This concern was dubbed “the tragedy of the anti-commons” (Heller, 1998), i.e., that exclusive rights may reduce innovation, or that such innovations do not reach all parts of society in a balanced way. The concerns have reached politics with the establishment of “Pirate Parties” in 38 countries with the main (or only) goal to “reform copyrights and related rights” (https://pp-international.net/about-ppi/, accessed July 2019).

Such debates may lead to either policy changes (adaptation of regulations or their implementation), or to novel uses of the rights. Open source strategies that have evolved to curb negative aspects of such exclusivity illustrate the latter approach.

This paper discusses an emerging open source movement in the plant breeding sector, dubbed “Open Source Seed.” We describe the two main “Open Source Seed” initiatives and discuss them on the basis of their primary call: to “free the plant genetic resources” from corporate controls. Will the initiatives indeed open the source further or will they create other bottlenecks, both for breeders not participating in the system. The second question is whether the initiatives will be able to curb the tragedy of the commons. Will OSSI provide for sufficient innovation for society at large that requires significant investments in plant breeding to contribute to societal goals, including through more robust plant varieties, consumption qualities, and reducing food waste.

We will then compare them to existing strategies and trends that either use IPR to avoid closing off of the source, the plant genetic resources, and others that aim at changing the regulatory systems.

## Open Source Seed Initiatives

### The OSSI-Pledge in the USA

The Open Source Seed Initiative (OSSI) was initiated in 2012 by an interdisciplinary team in Wisconsin, United States, “dedicated to maintaining fair and open access to plant genetic resources worldwide in order to ensure the availability of germplasm to farmers, gardeners, breeders, and communities of this and future generations.” (OSSI, 2016). The initiative is based on the analysis that only a handful of companies account for most of the world’s commercial breeding and sales of seed, and that patenting is a crucial tool in support of this trend by enhancing the power and control of these companies over the seeds and the farmers that feed the world (Kloppenburg, 2010).

Inspired by the open source software movement, OSSI wants to create a system that can go viral: initial plant materials would be freely available to breeders under the condition that the further use of any genetic resources (varieties) derived from them would be made available under the same “open source” conditions. In this way, the system would go viral up until the point that actors who would want to patent their work would not have many genetic resources left to base their breeding on—or at least that a strong parallel system would develop. OSSI specifically focuses on breeders, including farmer breeders. A difference with most open source initiatives in other sectors is these use the patent or copyright systems in order to increase openness. The holder of an IP right has the exclusive right on the commercialization of the invention (patent) or text (copyright). That right is conventionally used in a commercial setting where the IP portfolio can be a major asset of a company, but it can also serve to implement open source conditions down the chain: only users are admitted who follow the open source rules for the derivatives that they develop using the protected source material. The Intellectual Property thus allows the right holder to legally enforce such open source use. The OSS initiative in Wisconsin came to the conclusion that this would not be feasible for plant genetic resources and instead based its open source model on a non-legally-binding Pledge:

“You have the freedom to use these OSSI Pledged seeds in any way you choose. In return you pledge not to restrict others’ use of these seeds or their derivatives by patents or other means, and include this Pledge with any transfer of these seeds or their derivatives.” (https://osseeds.org/about/)

Even though this would not be legally enforceable, it creates a strong moral obligation. By doing that, it confirms with the opposition of the group against the increasing “juridification,” i.e., the increased influence of different laws, in breeding, and seed supply. It also wants to send out a strong message to society against the patenting trend in the seed sector.

In 2014, 37 varieties of 14 species were released by various public and private breeders under the OSSI-Pledge. Since the development of varieties takes several years, it is too early to judge whether the system is going viral on the basis of these first releases. Several plant breeders, notably those operating in the organic sector, and some in US universities, have followed suit.

### The OSS License—Germany

An initiative in Germany that builds on the OSSI example in the USA is taking a different approach (Kotschi and Horneburg, 2018). Where the Pledge is implemented on the basis of morality, the German initiative wants to rely on a legally enforceable bag-tag contract attached to each seed bag saying that opening the bag implies agreement with the conditions of the contract. This kind of contracts is widely used on products (shrink wrap) and websites (clickwrap), but their enforceability depends strongly on national laws. Several non-governmental organizations have argued in the past against the use of such contracts by large seed companies to restrict the use of the seeds by farmers (Organic Consumers Association, 2010). von Gierke (2016) analyzes bag-tag licenses under German law and concludes that there are many uncertainties connected to their enforcement.

The German initiative expects to be able to identify infringements of these contracts using modern genomics techniques, and through the implementation of the Nagoya Protocol. DNA sequence data could give an indication that an open source plant has been used in the breeding process, but its predictive power likely depends on the crop and the breeding methods used. The implementation of the Nagoya Protocol in Europe obliges breeders to be able to confirm legality of the genetic resources they have used to create a new variety. Breeders thus have to keep track of their use of genetic resources and their contracts with countries from where they obtained such parent materials. The prospective use in identifying infringements of open source contracts is based on the assumption that such pedigree information will be publicly available, which is currently not the case. The OSS License was first implemented in 2017 on an existing tomato variety bred by a university, and a wheat variety bred by a biodynamic breeder. Whereas the OSSI in the USA is based on a moral call for openness, the German initiative adds additional contractual obligations for breeders and thus further contributes to the juridification processes in the seed sector.

## Scope: Open Source or Seeds as a Common Good?

Open Source Seed claims to be an open source system for genetic resources. It does, however, not stop with making genetic resources (the source code) available for users to innovate further, but it also gives freedom to all to reproduce and sell seed of a particular variety that is available under the open source Pledge or License. Every farmer is free to reproduce a variety developed from open source germplasm and share/sell it to other growers; any seed supplier can offer the variety in the seed market. It is likely that such a seed producer can offer the same variety for a lower price than the original breeder, who has spent several years of work to develop the variety. The breeder will thus not likely recoup the years of investment needed to develop the new variety and has to have other resources to base his breeding work on. This goes beyond the concept of “freeing genetics” (Luby and Goldman, 2016) but proclaims a commons approach to seed both with breeders and farmers Kloppenburg, 2014).

The originators of the OSSI recognize that in, such a market, it will be difficult to generate profits that allow for substantial investment in plant breeding. In the USA, breeding of most crops is currently done in the public sector (the Land-Grant Universities). Even though such universities welcome income from their breeding operations just like commercial breeders, they may have good reasons to make products of their research available for free. In addition, OSSI expects voluntary contributions from seed users to provide options for sustaining breeding programs. Such payments may not be related to the agronomic benefits of the seed, but rather to their socio-political context. Luby et al. (2015) expect that contributions for “freed seed” would mirror willingness to pay higher prices for “fair trade” products. Osman et al. (2007) identify sharing responsibilities within the chain (supermarkets funding breeding) as a possible way to fund organic breeding (next to collaborating with commercial breeders). In Germany, where breeding is largely commercial, donations also—for example, through a “Saatgutfonds”^1^—support various local breeding initiatives. Such mechanisms have been developed quite recently. It remains to be seen whether they can sustain the long process of breeding.

## History of Openness in Breeding

### The Breeder’s Exemption

Openness has been an important point of discussion during the early decades of scientific plant breeding. Plant breeding was not included at the time that important concepts of the national patent systems were harmonized in Paris in 1883. Farmer breeding had been going on for millennia; commercial seed production had started a century before, but systematic breeding of field crops was a new development during the latter part of the 19^th^ century. Only after 1900, with the rediscovery of Mendel’s laws on heredity, breeding developed into a science. The effects of protection of industrial inventions triggered debates by breeders especially after the first World War (Heitz, 1987). This led in the USA to the Plant Patent Act in 1930, which was only applicable to vegetatively propagated crops with the exception of edible roots and tubers. That solution for ornamentals and fruits was not considered useful in war-torn Europe where food production needed to be stimulated, and better varieties were broadly considered essential. Germany initially used the copyright system to give breeders a commercial advantage as they would be exclusively allowed to put a seal on seed bags to identify “original seed” (Heitz, 1987). All other seed producers could copy the seed, but not the seal. In the Netherlands, a small levy was charged on quantities of certified seed potatoes, which funds were distributed among the breeders according to the acreage of seed potatoes of their varieties produced (van Leeuwen, 1957). The mark-up could not be significant since such would deter the use of quality-controlled seed and as such increasing the risks of spreading potato diseases. Such levy systems have been used by producing marketing boards all over the world.

All these systems would now be considered “open source.” They did, however, not provide a reasonable income to the breeder, which resulted in breeding remaining a hobby rather than a significant job, and that for the most important crops, the government took important responsibilities in many countries. Openness was, however, a major prerequisite when the IPR were discussed to support plant breeding. All *sui generis* systems that emerged in Europe and that were harmonized in the UPOV Convention in 1961 (www.UPOV.int) explicitly avoid that the (genetic re-)source can be privatized. One of the basic concepts of plant breeder’s rights is the breeders’ exemption, the right of all to freely use protected varieties for further breeding. In very few countries, such as the USA, patents can be obtained on plant varieties. The patent system does not have this open innovation character. The use for breeding of a plant that falls within the scope of a patent falls under the rights of the patent holder and requires a license. Since patenting of varieties is common in the USA; it is therefore understandable that the first OSSI emerged in that country.

The plant breeder’s rights systems are open with respect to the source (the genetic resource), but it does provide exclusive rights at the level of multiplication and sales of seeds of the protected variety. That rule is essential for creating a business model for plant breeding. In Europe, an estimated 15% of the sales of seed is invested in breeding. This signifies a considerable societal benefit as breeding is focused mainly on i) disease resistance as major strategy to reduce crop losses and the use of crop protection chemicals; ii) stress tolerance, currently increasingly important at the time of climate change and its associated risks for farmers; and iii) product quality, including taste and shelf life, reducing food losses.

The OSSIs extend the openness to all who want to multiply the “open source seeds.” They do not only keep the source open but also give rights to those who do not innovate, but merely copy the variety. This creates additional competition in the seed market by those who have not invested in breeding similar to the situation in Europe before the variety protection systems.

### Restricting Openness: Patents and Biodiversity Rights

With the developments in biotechnology, the patent system entered plant breeding. That system is rooted in the industrial business culture as opposed to the agri-cultural origins of plant breeder’s rights. Patents can be granted to breeding processes and products. In most countries, essentially biological processes are exempted based on the TRIPS Agreement of the WTO (https://www.wto.org/english/tratop_e/trips_e/intel2_e.htm). Most countries, with the exception of the USA, also exempt plant varieties from patentability. However, plant traits are in most cases patentable when they meet the general patent criteria of novelty, non-obviousness/inventive step, and industrial use. The patent system does not have a breeder’s exemption. Doing experiments with the invention in order to create a new commercial product requires a license of the patent holder. The holder of a patent on one trait could thus stop a breeder from using that plant for further breeding. The patenting of plant traits and its impact on the breeder’s exemption worries breeders (see below).

Furthermore, countries have national sovereign rights over genetic resources as of 1993 (CBD, 1993) including crops. Countries, notably those in the so-called centers of origin (Vavilov, 1951) or centers of diversity (Hawkes, 1983) can make access to such genetic resources by breeders and researchers subject to “Prior Informed Consent” and “Mutually Agreed Terms.” The Convention on Biological Diversity (CBD) (https://www.cbd.int/convention/text/) charged countries to identify national competent authorities to manage such access negotiations. Terms of such contracts may affect not only the primary user but also downstream users of the genetic resource, depending on national law. The Nagoya Protocol under the CBD (CBD, 2011) describes user obligations and charges authorities in user countries to control adherence to the contracts. An awkward thing of that Protocol is that copying a genetic resource is allowed, but innovating with it is not. These rules are getting increasingly complex with time as more and more genetic resources that breeders want to use in their crossing programs would fall under the rules.

Open innovation is, thus, not only restricted by patents but it is also increasingly challenged by biodiversity rights.

## Other Open Source Initiatives

Open source initiatives are many. The most common are open source software and open source publishing. The former includes the Linux Community (https://www.linux.org/) and the Chromium Projects (https://www.chromium.org/) inviting all to study and contribute to software improvements based on publicly available source code. These were initiated as a response to the dominance of Microsoft. The open source software initiatives keep the source code open and stimulate software developers to create solutions for particular uses. Such programs may, however, be commercialized. Open source publishing aims at the freedom to copy materials, which is quickly gaining popularity in scientific publishing. It is not the reader (or the library) but the author who is charged to cover the cost of publishing.

The breeder’s rights system actually illustrates that model: the source is open and the user pays (through a mark-up on the seed price).

An initiative close to the OSSI was initiated in 1992 by the social enterprise CAMBIA (https://cambia.org/). This was an initiative by Richard Jefferson, one of the inventors of a critical component of biotechnology—the GUS-reporter system. He undertook several attempts to “democratize invention” in biotechnology, as it was considered stifled by the patent system that had grown so complex that—according to him—only few experts understand how it operates and how to decipher the treasures hidden in patents. CAMBIA developed the Patent Lens in order to increase transparency in patent landscapes (https://www.lens.org/) and worked on improving rice using new technologies. Inventions were patented and initially licensed out at very favorable terms to users working for the public good and at commercial terms to corporate users. Alternatives to the commonly used *Agrobacterium* transformation system were developed, thus bypassing the main genetic transformation patents (Broothaerts et al., 2005). BiOS, “Biology Open Source” (https://cambia.org/bios-landing/), was established as an initiative to make alternatives for every step in the modification process available under an open source license. However, this did not lead to a significant reduction in the use of commercial licenses on the *Agrobacterium* technologies. However, the initiatives did and still do impact debates on innovation in plant science.

## Other Ways to Limit Negative Effects of Patents

### Reduce the Scope of Patents

The patenting of plants has been a concern of many stakeholders, including the plant breeding community itself. Breeders in Europe have argued for more than a decade that the lack of a breeder’s exemption in the patent system can cause serious limitations to breeders unless they are willing to spend significant resources in legal counsel by patent specialists. In 2009, the Dutch Seed Association Plantum called for an inclusion of a breeder’s exemption in patent law. This triggered debates both in the breeding community, in parliaments and among legislators. The European Union decided in 2014 to include a “limited breeder’s exemption” in the new Unitary Patent System. This means that breeders are free to use plant materials that contain patented traits, and only when the new variety that they develop contains that patented trait, they will need to have a license from the patent holder to commercialize the variety.

### Limit Patentability

The same debate also yielded another change. The Council of EU Ministers unanimously voted in favor of a Commission Notice, interpreting the EU Directive 98/44 with the effect that patents on natural traits (“products of essentially biological processes”) should not be granted (EU, 2016). Implementation of that decision by the European Patent Office proves more complex than anticipated. An attempt by the Office to implement the wish of its Council using Rule 28, exceptions to patentability is challenged at the time of writing this paper.

### Patent Pools

A way to soften major negative effects of patenting, patent pools can also be a strategy. An example is the “International Licensing Platform—vegetables” (www.ilp-vegetable.org), and initiative by major vegetable-breeding companies. The agreement under the ILP is that all requests for a license have to be honored, i.e., access to a patented invention cannot be withheld by the patent holder. A referee system was put in place to determine fair license conditions when the two parties cannot conclude a contract within a given time period. It is framed as “free access but not for free.” Whether this is a solution for all breeders remains to be seen.

## Analysis

The first important effect of the open source seed movements is that it contributes to the debate about innovation systems and the place of different types of intellectual property in search of maximizing societal benefits and minimizing monopolistic behavior. IPR have been developed in order to stimulate invention and intend to support the actual use of inventions in practical innovations that better our lives. Finding a balance between the rights and obligations of the inventor is complex in a society where quickly advancing technology increasingly puts the inventor at a distance from society at large.

An important question would therefore be whether doing away with such rights would result in more and better innovations. Could public funding provide the foundation for all plant breeding? Or could alternative funding mechanisms provide sufficient funds sustainably, which is required to develop better varieties of all different crops for the diversity of farming systems and for a quickly changing society? Lammerts van Bueren et al. (2018) and Kotschi and Wirz (2015) identify the challenge and suggest a combination of public and chain partner (through foundations) funding. Looking at the vast investments made in plant breeding—in the Netherlands vegetable-breeding sector alone, conservatively estimated at 300 million Euro per year—it may be doubted that the same intensity of breeding could be sustainably supported through such funding mechanisms. Here, it is important to identify the difference between the concept of “open source,” which applies to the use of genetic resources, and “commons” which also relates to the use of seeds. The literature on the subject does not make that distinction. The OSS initiatives highlight the former but appear to pursue the latter.

Another effect of the open source license is likely, in which when it would operate side-by-side commercial breeding, it would add again to limiting the breeder’s exemption. A breeder who operates in the business models of the open innovation system created by breeder’s rights could not “touch” any material under the OSS license without challenging his freedom to protect the new varieties that he would produce. The trend created by patents and biodiversity rights of limiting access by breeders to the genetic diversity that they need to breed better varieties would be attacked from yet another side. Openness can thus limit access to genetic resources just like patents and biodiversity rights. Initiatives that simply do not apply for patents or breeder’s rights (Wirz et al., 2017) would not have such negative side effects. Such public domain approaches are not popular in the open source literature as it may invite others to appropriate “derivatives” (Luby et al., 2016).

Alternative ways to rebalance the rights with the benefits of society through regulatory change require significant time. This is likely less when implementing rules are changed compared to adapting the laws themselves. Rebalancing through voluntary measures by right holders themselves has the disadvantage that not all parties may join. From an open source perspective, the breeder’s rights system creates no issues.

## Conclusion

“Open Source Seed” responds to an ongoing debate in society about the provision of IPR that, on the one hand, aim at supporting innovation or creative works, but that also risks to stifle the same through its exclusive rights.

It is understandable that the OSSI emerged in the United States, where patenting of plant varieties is common, and internationally agreed rights on biological diversity are not valid. The USA is unique in this legal position.

If the movement is also meant to oppose the “juridification” of plant breeding, then the OSSI-Pledge used in the USA is a more logical, but legally less strong, solution that the OSS license proposed in Germany.

If it is the intent to create openness toward genetic resources, then the breeder’s exemption in the plant breeder’s rights system, which is the dominant protection system in almost all countries already, fulfils these needs. When, however, it is not only the intent to protect the (genetic re-) source but also to allow everybody to compete in the seed market with breeders, then the name “open source” is a misnomer. Then, the initiatives may be better framed under the concept of the Commons. But that term would contradict the effect that the OSS Initiative, at least the one operating through the license system, can reduce access to genetic resources by conventional plant breeders in their business model.

There are alternative ways to limit the impact of patent rights, i.e., by rebalancing the patent system itself and by avoiding strategic (monopolistic) use of the rights through patent-pool type of agreements. Neither these, nor the open source seed initiatives, can reduce the negative impact of biodiversity rights on the openness of genetic resources for plant breeding.

## Author Contributions

The author confirms being the sole contributor of this work and has approved it for publication.

## Conflict of Interest Statement

The reviewer [EL] declared a shared affiliation, with no collaboration, with the author to the handling editor at the time of review. The author declares that the research was conducted in the absence of any commercial or financial relationships that could be construed as a potential conflict of interest. Since the author is connected to the association of seed companies in The Netherlands, almost exclusively operating in the commercial seed market, some may consider that the article that is critical of the open source seed concept may be informed by this association. The author has however published articles that are critical to intellectual property in his career, he has not involved any of the member companies in this writing project, and he has no immediate interest in selling seed (so no personal interest in or against the open source movement).

## Footnotes

1 https://www.zukunftsstiftung-landwirtschaft.de/saatgutfonds/infomaterial/aktueller-infobrief-saatgutfonds/
